# The pattern of disorders of sex development in Vietnamese children

**DOI:** 10.1186/1687-9856-2015-S1-P115

**Published:** 2015-04-28

**Authors:** Pham Thu Nga, Vu Chi Dung, Bui Phuong Thao, Nguyen Ngoc Khanh, Can Thi Bich Ngoc, Hoan Nguyen Thi, Dat Nguyen Phu

**Affiliations:** 1 Hanoi Medical University, Hanoi, Vietnam; 2 Vietnam National Hospital of Pediatrics, Hanoi, Vietnam

**Keywords:** Disorders of sex development, DSD, 46,XX DSD, 46,XY DSD, CAH.

## Background

Disorders of sex development (DSD) are defined as congenital condition in which development of chromosomal, gonadal, or anatomical sex is atypical. The Chicago DSD classification includes three main diagnostic categories: sex chromosome DSD, 46,XY DSD and 46,XX DSD.

## Aims

Define the pattern of disorders of sex development according to Chicago’s classification 2006 at National Hospital of Pediatrics in Hanoi, Vietnam (NHP).

## Method

Patients were examined, diagnosed and treated DSD or ambiguous sex at (NHP) from 31/07/2002 to 31/7/2012. Criteria that suggest DSD include

1. overt genital ambiguity (eg, cloacal exstrophy)

2. apparent female genitalia with an enlarged clitoris, posterior labial fusion, or an inguinal/labial mass

3. apparent male genitalia with bilateral undescended testes, micropenis, isolated perineal hypospadias, or mild hypospadias with undescended testis

4. a family history of DSD such as CAIS, and

5. a discordance between genital appearance and a prenatal karyotype. Method of the study was descriptive observational.

## Results

51.7% patients had 46,XX DSD, among them 98.6% had definitive diagnosis. Congenital adrenal hyperplasia (CAH) is the most common cause of 46,XX DSD (91.9%). Rate of 46,XY DSD was 25%, however 83.3% had no definitive diagnosis. 23.3% of patients had chromosome DSD, among them 88.3% chromosome DSD was Turner syndrome.

## Conclusion

CAH is the most common cause of DSD.

